# Evaluate the Fatigue Life of CFRC Subjected to Coupled Thermo–Mechanical Loading

**DOI:** 10.3390/ma12182886

**Published:** 2019-09-06

**Authors:** Junjie Ye, Wangpeng He, Yang Shi, Yiwei Wang, Gaigai Cai, Zhi Zhai, Xuefeng Chen

**Affiliations:** 1 Research Center for Applied Mechanics, Key Laboratory of Ministry of Education for Electronic Equipment Structure Design, Xidian University, Xi’an 710071, China; 2 School of Aerospace Science and Technology, Xidian University, Xi’an 710071, China; 3 State Key Laboratory for Manufacturing Systems Engineering, Xi’an Jiaotong University, Xi’an 710049, China

**Keywords:** fatigue life, temperature, polymer composites, stress distribution

## Abstract

Mechanical properties of composites manufactured by high-temperature polymer polyether ether ketone (PEEK) with continuous reinforced fibers are closely dependent on ambient temperature variations. In order to effectively study fatigue failure behaviors of composites under the coupled thermo–mechanical loading, a well-established microscopic model based on a representative volume element (RVE) is proposed in this paper. Stiffness degradation behaviors of the composite laminates at room and elevated temperatures are firstly investigated, and their failure strengths are compared with experimental data. To describe the fatigue behaviors of composites with respect to complex external loading and ambient temperature variations, a new fatigue equation is proposed. A good consistency between theoretical results and experimental data was found in the cases. On this basis, the temperature cycling effects on the service life of composites are also discussed. Microscopic stress distributions of the RVE are also discussed to reveal their fatigue failure mechanisms.

## 1. Introduction

Due to their excellent mechanical properties [1,2], such as fatigue resistance, good thermal stability, and fracture toughness [3,4], high-temperature polymer composites have been widely used in the aviation industry, and they are looking to replace metal materials in a variety of aircraft components, such as luggage racks and cable clips [5]. Airbus has discussed and intended to use high-temperature composite structures for wing structures and thicker fuselage structures [6]. 

In recent years, thousands of researchers have devoted themselves to deeply investigating the fatigue properties of composites by using experimental or theoretical methods [7,8,9,10]. Seyfullayev [7] and Li [8] established a model to investigate fatigue crack growth in composite structures. Carvalho et al. [8] studied the influence of reinforcement mechanisms of carbon nanotubes on wear, as well as fatigue tests on an aluminum–silicon composites. Huang et al. [10] employed the experimental method to study crack bridging in engineered cementitious composites under fatigue tensile loading. The fatigue properties have also been observed as the change in residual strength, cracks, external loading, stiffness, etc. [11,12,13,14,15]. Huang [11] and Zhao [12] established effective models to analyze the degradation properties and progressive fatigue damage in composite structures under fatigue tensile loading. At several temperatures, Eftekhari and Fatemi [13] investigated the cycling frequency on fatigue behaviors by conducting load-controlled fatigue tests. The results showed that fatigue behavior is also time-dependent for frequency-sensitive materials. Keulen et al. [14] presented a novel technique to predict the remaining life of composites under fatigue loading by employing the embedded fiber Bragg grating sensors. Based on experimental observations, fatigue cracks were investigated and considered to first appear in a matrix for UD composites with respect to tensile–tensile cycling loadings. Furthermore, they accumulate and expand in the interphase [15] and reinforced fibers [16]. Garcea et al. [17] employed synchrotron X-ray computed tomography to study fiber fatigue failures in carbon/epoxy composites. The accumulation and distribution of fiber breaks were effectively evaluated. Pakdel and Mohammadi [18] made an experimental test to investigate the onset of crack saturation in matrix materials and mid-ply matrix cracks in laminates. On this basis, the saturation crack densities subject to fatigue loadings were further investigated. It should be pointed out that that experimental procedures needs huge investment in terms of cost and time. Therefore, some researchers prefer to study fatigue failure behaviors by establishing a proper model. Alves and Pimenta [19] proposed a micromechanical analytical model to predict the fatigue life of unidirectional composites under tension–tension loading. Model availability were successfully validated by experiment data from the literature. Hamidi et al. [20] investigated the fatigue behaviors of thick composite laminates under flexural loading, and good agreement between the experimental data and theoretical results was obtained.

Composite structures are always exposed to different ambient temperatures in service [21]. Yuan et al. [22] studied the damping capacity of composites at a high temperature. Nguyen and Nghiem [23] investigated the buckling behaviors of a composite plate under an unsteady temperature field. It is no doubt that material properties are always temperature dependent. In general, a higher ambient temperature will decrease the failure strength and stiffness behavior of most kinds of materials. Correspondingly, the fatigue properties of composites under different ambient temperatures may be a disastrous problem. Some investigators documented the fatigue life for high-temperature polymer composites. Campa [24] focused on the cryogenic fatigue life of composite structures under cyclic strain. Preliminary experimental results indicated that the cryogenic fatigue life is at least 20 times longer than at room temperature. Yang and Yang [25] established a model to investigate the thermal fatigue life of SiCp/A356 composites. The numerical results obtained agreed well with the experimental data. Godin et al. [26] acquired lifetime diagrams of ceramic matrix composites below 600 °C, and fatigue damage evolution was monitored by experimental methods of strain measurement and acoustic emission. Johnston [27] investigated the fatigue behaviors of continuous fiber-reinforced ceramic matrix composites in a range of temperatures from 20 to 1200 °C. The role of stress redistribution interactions between fibers and matrix were considered. Pandey and Arockiarajan [28] developed a theoretical model consisting of non-linear finite analysis integrated with the cumulative damage theory to predict the fatigue behaviors of composites. Moreover, the method was further extended to investigate stiffness degradations of composites under various thermo–mechanical loadings. The fatigue analysis mentioned above is limited to room temperature or elevated temperatures. A combination of fatigue loading and cycling temperature will lead to synergistic effects on the fatigue degradation of composite structures in service. It is a challenging topic to reveal fatigue failure mechanisms and predict fatigue life under a coupled thermo–mechanical loading. A compressive study referring to an evaluation of the fatigue properties of composites under coupled thermo–mechanical loading was executed by Eftekhari and Fatemi [29]. However, few studies concern the microscopic stress distributions of fiber composites subjected to coupled thermo–mechanical loading.

This paper aimed to grasp the fatigue behaviors of composites with respect to coupled thermo–mechanical loading. To this end, a new set of fatigue equations based on computational micromechanics was proposed. After determining the fatigue parameters according to the *S-N* curves of resin matrix and composites, the proposed model was employed to evaluate the fatigue life under coupled thermo–mechanical loading. Furthermore, the microscopic stress distributions in the representative volume element (RVE) was also indicated to reveal failure mechanisms at microscopic scale. 

## 2. Theoretical Aspects

As one of the heterogeneous materials, multi-mode damages, such as fiber breakage [30], matrix [31], and interfacial cracks [32,33], are coupled and influence each other in composites. In addition, composite structures may be subjected to cycling temperature loading. Service life and microscopic failure investigations under coupled thermo–mechanical loading will lay a solid foundation for their usage and optimized structural design. Therefore, an effective microscopic model is urgently needed. To simplify the modelling procedure at the microscopic scale, herein, it was assumed that reinforced fibers are periodically distributed in matrix materials, and composites are composed of an infinite number of representative volume elements. On this basis, the RVE is further divided into $N_{\beta }*N_{\gamma }$ sub-cells. According to the homogenization theory, the displacement vectors, $u_{i}(x,y)$, can also be expressed as the function of the average displacement term, $u_{i}^{\left(0\right)}(x,y)$, and their high-order terms, $u_{i}^{\left(n\right)}(x,y)(n=1,2,\ldots )$, that is:

(1) $$u_{i}(x,y)=u_{i}^{\left(0\right)}(x,y)+\delta u_{i}^{\left(1\right)}(x,y)+O(\delta^{2}),$$

where $x=(x_{2},x_{3})$ and $y=(y_{2},y_{3})$ are the macroscopic and microscopic coordinate systems, respectively.

Due to its periodicity hypothesis of the RVE mentioned above, the boundary continuity condition of displacement terms, $u_{i}^{\left(n\right)}(x,y)$, can be further expressed as:

(2) $$u_{i}^{\left(n\right)}(x,y)=u_{i}^{\left(n\right)}(x,y+k),$$

where k indicates the periodic dimension of the RVE at the microscopic scale. 

It should be noted that the average displacement, $u_{i}^{\left(0\right)}(x,y)$, is unrelated to material parameters at the microscopic scale, and the component can be simplified as $u_{i}^{\left(0\right)}(x)$. Therefore, the displacement vectors, $u_{i}(x,y)$, in Equation (1) can be rewritten and expressed as follows:

(3) $$u_{i}(x,y)={\bar{u}}_{i}(x)+{\tilde{u}}_{i}(x,y)+O(\delta^{2}),$$

where ${\tilde{u}}_{i}=\delta {\tilde{u}}_{i}^{\left(1\right)}$. $O(\delta^{2})$ indicates the higher-order terms of the fluctuating displacements.

According to the elastic theory, average strain components, ${\bar{\varepsilon }}_{ij}(x)$, and fluctuating strain components, ${\tilde{\varepsilon }}_{ij}(x,y)$, can easily be expressed as a function of displacement vectors, ${\bar{u}}_{i}$, and their high-order terms, ${\tilde{u}}_{i}$, that is:

(4) $${\bar{\varepsilon }}_{ij}(x)=\frac{1}{2}\left(\frac{\partial {\bar{u}}_{i}}{\partial x_{j}}+\frac{\partial {\bar{u}}_{j}}{\partial x_{i}}\right),$$

(5) $${\tilde{\varepsilon }}_{ij}(x,y)=\frac{1}{2}\left(\frac{\partial {\tilde{u}}_{i}}{\partial y_{j}}+\frac{\partial {\tilde{u}}_{j}}{\partial y_{i}}\right).$$

Combining Equation (3) with Equations (4) and (5), the strain components, $\varepsilon_{ij}(x,y)$, in composites can be expressed as follows:

(6) $$\varepsilon_{ij}(x,y)={\bar{\varepsilon }}_{ij}(x)+{\tilde{\varepsilon }}_{ij}(x,y)+O(\delta^{2}).$$

Based on the strain relations shown in Equation (6), as well as the modelling scheme of the high-fidelity generalized method of cells (HFGMC), the relations between sub-cell displacements, $W^{\left(\beta \gamma \right)}=\left[u_{1}^{\left(\beta \gamma \right)}\;u_{2}^{\left(\beta \gamma \right)}\;u_{3}^{\left(\beta \gamma \right)}\right]$, and average sub-cell displacements, $W_{\left(00\right)}^{\left(\beta \gamma \right)}=\left[{\bar{u}}_{1}^{\left(\beta \gamma \right)}\;{\bar{u}}_{2}^{\left(\beta \gamma \right)}\;{\bar{u}}_{3}^{\left(\beta \gamma \right)}\right]$, in a local coordinate system can be written as [34]:

(7) $$W^{\left(\beta \gamma \right)}=\bar{W}+W_{\left(00\right)}^{\left(\beta \gamma \right)}+y_{2}^{\left(\beta \right)}W_{\left(10\right)}^{\left(\beta \gamma \right)}+y_{3}^{\left(\gamma \right)}W_{\left(01\right)}^{\left(\beta \gamma \right)}+\frac{1}{2}(3y_{2}^{(\beta )2}-\frac{h_{\beta }^{2}}{4})W_{\left(20\right)}^{\left(\beta \gamma \right)}+\frac{1}{2}(3y_{3}^{(\gamma )2}-\frac{l_{\gamma }^{2}}{4})W_{\left(02\right)}^{\left(\beta \gamma \right)},$$

where $h_{\beta }^{}$ and $l_{\gamma }^{}$ are the sub-cell height and length in the RVE, respectively. $W_{\left(pq\right)}^{\left(\beta \gamma \right)}$ indicates higher-order displacement terms.

Similarly, temperature components in composites can be asymptotically expanded as the function of the small scaling parameter, δ, when the steady heat transfer problem is considered, that is:

(8) $$T(x,y)=T^{\left(0\right)}(x)+\delta T^{\left(1\right)}(x,y)+\delta^{2}T^{\left(2\right)}(x,y)+\cdots ,$$

where T and $T^{\left(0\right)}$ indicate temperature and average temperature components, respectively. $T^{\left(n\right)}(n=1,2,\cdots )$ indicates the high-order terms of temperature components. 

According to Fourier’s law, the relations between sub-cell heat flux vectors, $q_{i}^{\left(\beta \gamma \right)}$, and thermal conductivity coefficients, $k_{ij}^{\left(\beta \gamma \right)}$, can be written as follows:

(9) $$q_{i}^{\left(\beta \gamma \right)}=-k_{i}^{\left(\beta \gamma \right)}\frac{\partial T^{\left(\beta \gamma \right)}}{\partial x_{i}}=k_{i}^{\left(\beta \gamma \right)}H_{i}^{\left(\beta \gamma \right)},$$

where $H_{i}^{\left(\beta \gamma \right)}$ is related to the sub-cell temperature gradient. 

For investigating the thermal conductivity problem of the CFRC under steady-state conditions, the local equilibrium equation in the RVE can be expressed as:

(10) $$-\frac{\partial q_{i}^{\left(\beta \gamma \right)}}{\partial x_{i}^{}}=0.$$

Similar to solving procedures of the sub-cell displacements, sub-cell temperature components, $T^{\left(\beta \gamma \right)}$, will be expressed as a function of the heat flux vectors, $q_{i}^{\left(\beta \gamma \right)}$, and thermal conductivity coefficients, $k_{ij}^{\left(\beta \gamma \right)}$. To solve the coupled thermo–mechanical problems, sub-cell displacement components, $W^{\left(\beta \gamma \right)}$, were further extended as a function of the sub-cell displacements and temperatures, that is:

(11) $$W_{e}^{\left(\beta \gamma \right)}=\left[{\tilde{u}}_{1}^{\left(\beta \gamma \right)}\;{\tilde{u}}_{2}^{\left(\beta \gamma \right)}\;{\tilde{u}}_{2}^{\left(\beta \gamma \right)}\;T_{}^{\left(\beta \gamma \right)}\right].$$

In the procedure of thermo-mechanical modelling, the constitutive relation of the CFRC is employed and the sub-cell average stress, ${\bar{\sigma }}_{ij}^{\left(\beta \gamma \right)}$, and heat flux vectors, $q_{i}^{\left(\beta \gamma \right)}$, can be expressed as functions of the sub-cell strains, ${\bar{\varepsilon }}_{ij}^{\left(\beta \gamma \right)}$, and temperature gradients, $H_{i}^{\left(\beta \gamma \right)}$. In order to simplify the calculation and improve efficiency, the surface average displacements, tractions, and heat flux in each sub-cell were employed. In addition, the continuity between adjacent sub-cell displacements and tractions was employed to solve the coupled thermo–mechanical equations. Detailed modelling procedures were reported by Aboudi and Ye [34,35]. Finally, the macroscopic average component, $\bar{Y}={\left[{\bar{\sigma }}_{11}^{}\;{\bar{\sigma }}_{22}\;{\bar{\sigma }}_{33}\;{\bar{\sigma }}_{23}\;{\bar{\sigma }}_{13}\;{\bar{\sigma }}_{12}\;q_{1}\;q_{2}\;q_{3}\right]}^{T}$, can be expressed as a function of the macroscopic average strains, ${\bar{\varepsilon }}_{ij}(i,j=1,2,3)$, and temperature gradients, $H_{i}(i=1,2,3)$, according to the homogenization theory, that is:

(12) $$\bar{Y}=\frac{1}{hl}\sum_{\beta =1}^{N_{\beta }}\sum_{\gamma =1}^{N\gamma }h_{\beta }l_{\gamma }A^{\left(\beta \gamma \right)}X,$$

where $X=\left[{\bar{\varepsilon }}_{11}\;{\bar{\varepsilon }}_{22}\;{\bar{\varepsilon }}_{33}\;{\bar{\varepsilon }}_{23}\;{\bar{\varepsilon }}_{13}\;{\bar{\varepsilon }}_{12}\;-H_{1}\;-H_{2}\;-H_{3}\right]$. $A^{\left(\beta \gamma \right)}$ indicates a 9×9 matrix. 

## 3. Materials and Work Method

### 3.1. Materials

#### 3.1.1. Specimen Preparations

**Figure 1:**
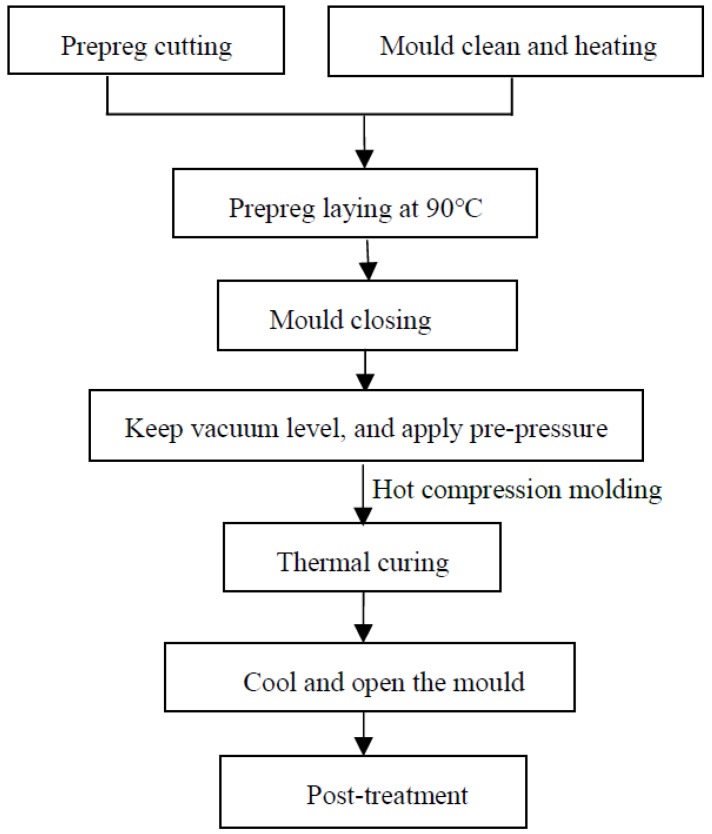
Manufacturing procedures of composite specimens.

In general, material properties are closely dependent on the ambient temperature. It means that their parameters, such as elastic constants and effective strength, are temperature dependent. To analyze the effective behaviors and fatigue properties of AS4/PEEK composites (Xiechuang Composite Materials Co. LTD, Dongguan, China) subjected to coupled thermo–mechanical loading, constituent material parameters were considered to be a function of the ambient temperature. The specimens were prepared by stacking and cutting the composite laminates, and their dimensions were determined according to the standard of ASTM D3039/D3039M [36] and STP1330-98 [37]. The manufacturing procedures of composite specimens, which are shown in Figure 1, can be summarized as the following steps:

(1) Cut and lay the carbon fiber prepreg according to the desired sequences of composite laminates.
(2) Clean mold and apply the release agent uniformly on it. In this procedure, we should try to keep the mold surface as smooth as possible.
(3) Raise the ambient temperature in the mold to 90 °C, and then open the mold. Transfer the layered prepreg to the mold cavity.
(4) Close the mold and start to vacuum. To acquire composite structures with a high quality, the inner vacuum degree should be kept at around −97 KPa. Moreover, a pre-pressure of 0.5 MPa is applied and kept for 5 to 15 s before the thermal curing process.
(5) The inner pressure is raised to 8 to 10 MPa, and the inner temperature remains at 200 ± 3 °C. This thermal curing process lasts about 30 to 90 s.
(6) Turn off the vacuum apparatus and heat source. When the temperature drops down to 80 °C, the product can be easily demolded and taken out. To acquire standard specimens of composites, the Delong DX25S machine tool (Delong CNC equipment Co. LDT, Taizhou, China) with emery wire was employed.

#### 3.1.2. Matrix Material Properties

Table 1 indicates the matrix material properties of the Polyetheretherketone (PEEK, Victrex plc, Lancashire, UK) at room temperature (23 °C) and elevated temperatures. It is clearly indicated that material properties are temperature dependent. A higher ambient temperature tends to further reduce the elastic modulus and effective strength. The glass transition temperature of the PEEK resin is 143 °C. Composites with continuous fibers AS4 (Hercules company, Wilmington, DE, USA), which afford excellent high-temperature properties, have good chemical resistance.

### 3.2. Work Method

#### 3.2.1. Tensile Test

**Figure 2:**
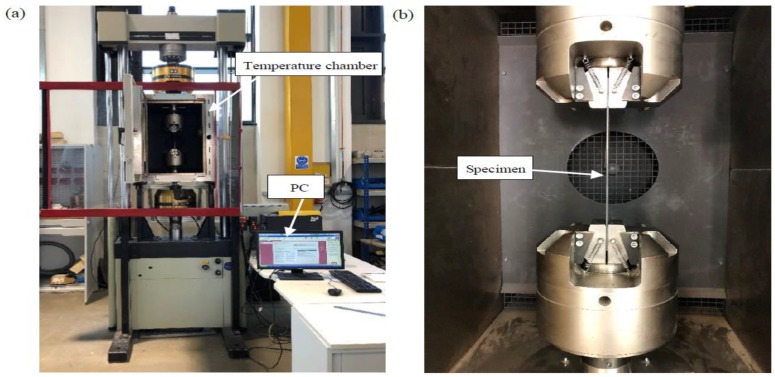

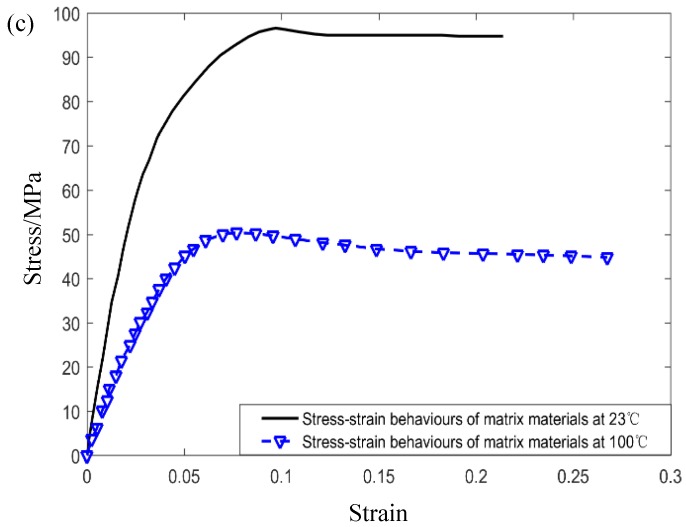
Mechanical behaviors test at room and elevated temperatures. (**a**) MTS. (**b**) Fixture and specimen. (**c**) Matrix materials at room and elevated temperatures.

In the tensile test procedure, the test system was composed of Material Test System (MTS, Instron 8802J5219, 250NK, Norwood, MA, USA) and the PC is shown in Figure 2a,b. The temperature chamber (Instron CP100358, maximum temperature 350 °C, Norwood, MA, USA) was adopted to control and keep the ambient temperature at specified levels, i.e., room temperature and 100 °C, during the test. Before tensile tests at the elevated temperature, it was kept for 3 min when the temperature increased to 100 °C in order to control the uniform distribution over the cross-section of the specimens. For each case, three specimens were employed. The stress–strain behaviors of the PEEK matrix under a strain rate of $2\times 10^{-5}$ are seen in Figure 2c. The experiment results indicate that the PEEK matrix at room and high temperatures presents high ductility. However, a higher ambient temperature tends to sharply decrease the loading capacity. In detail, the longitudinal tensile strength of the PEEK matrix was 94.58 and 50.37 MPa when room temperature and 100 °C were considered, respectively.

A proper RVE plays an important role in studying the mechanical properties of composite structures. In order to verify the effectiveness of the microscopic model, two typical ply methods of ${\left[0/90\right]}_{4s}$ and ${\left[0^{{}^{\circ}}/+45^{{}^{\circ}}/90^{{}^{\circ}}/-45^{{}^{\circ}}\right]}_{2s}$ composite laminates were considered in the cases. The material parameters of AS4 fibers, such as elastic moduli, Poisson’s ratio, and failure strength, were provided by Soden et al. [38,39,40,41]. It can be seen that the PEEK matrix was considered to be isotropic. In this material system, the fiber volume fraction (VF), which was determined by using the micrograph of the fiber cross-section, is approximate to 0.60. It should be pointed out that the shear strength of PEEK at 100 °C was acquired by employing the linear interpolation method based on the failure properties of the PEEK at room temperature [41] and a high temperature of 90 °C derived from the experimental methods [42]. Table 2 indicates a comparison of the longitudinal moduli of composite laminates between the experimental data and theoretical results at both room temperature and high temperatures. In a word, a high prediction accuracy of the effective moduli can be obtained. The maximum relative error is approximate to 2.90% when composite laminates ${\left[0^{{}^{\circ}}/+45^{{}^{\circ}}/90^{{}^{\circ}}/-45^{{}^{\circ}}\right]}_{2s}$ under room temperature are considered.

**Figure 3:**
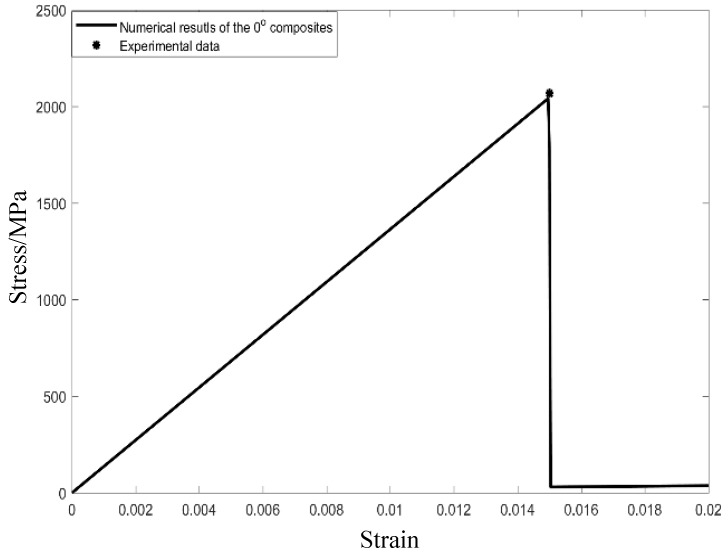
Stress–strain curve of the $0^{{}^{\circ}}$ lamina at room temperature.

To further confirm the rationality of this chosen RVE, the stiffness degeneration analysis and failure strength comparisons of composite laminates were also examined. To describe constituent material damages at room and elevated temperatures, the 3D Tsai-Hill and Maximum stress criteria [43,44] were developed and proposed to study microscopic damages in the matrix and fibers, respectively. Figure 3 indicates the stress–strain of the lamina with $0^{{}^{\circ}}$-reinforced fibers at room temperature. A linear stress–strain behavior can easily be found until the longitudinal strain approximates to 1.5%. Moreover, the maximum stress acquired by the theoretical modelling and experimental method are 2041.1 and 2068.0 MPa, respectively. It means that the relative error of the theoretical method is less than 2% compared with the experimental data.

**Figure 4:**
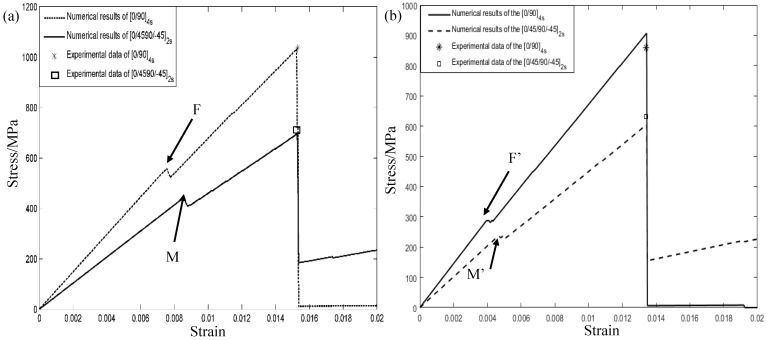
Stress–strain curves of the composite laminates: (**a**) 23 °C; (**b**) 100 °C.

Based on validations of the micromechanical method in analyzing the lamina, herein, stress–strain properties of ${\left[0/90\right]}_{4s}$ and ${\left[0/45/90/-45\right]}_{4s}$ composite laminates were also considered. To this end, the implemented procedures were executed by employing the microscopic model at each integration point of the lamina along the thickness direction [40]. The longitudinal tensile strength of the fibers was assumed to be 2900 MPa at 100 °C. The stress–strain results of composite laminates at room and elevated temperatures are shown in Figure 4. It can be seen that the laminates ${\left[0/90\right]}_{4s}$ always provide higher stiffness behaviors and final failure strengths than the laminates ${\left[0/45/90/-45\right]}_{4s}$ whether the ambient temperature is considered or not. Compared with the unidirectional (UD) lamina as show in Figure 3, obvious differences in the stress–strain behaviors were found when the layer sequence was considered in the example. In detail, obvious turning points, F and M, in the stress–strain curves of composite laminates at room temperature can easily be found as shown Figure 4a. Axial tensile loading tends to linearly increase their stress–strain behaviors at the initial stage. However, a sharp downward phenomenon was found when longitudinal strains increased to 0.76% for the ${\left[0/90\right]}_{4s}$ composite laminates. Combined with the mechanical properties of the lamina acquired by Ye et al. [40], these interesting results are attributed to the fact that the $0^{{}^{\circ}}$ lamina always provides the higher failure strength compared to the $45^{{}^{\circ}}$ and $90^{{}^{\circ}}$ laminas. In other words, the $0^{{}^{\circ}}$ fibers continue to sustain the external loading after the $45^{{}^{\circ}}$ and $90^{{}^{\circ}}$ laminas’ failure. Similar explanations refer to the turning points, F′ and M′, as shown in Figure 4b, which can be used when the ambient temperature increases to 100 °C. For a comparison, experimental data of the failure strength at 100 °C acquired by Jen et al. [45] are also indicated. It can be easily found that the theoretical predictions tend to overestimate and underestimate their final failure strengths of ${\left[0/90\right]}_{4s}$ and ${\left[0/45/90/-45\right]}_{4s}$ composite laminates to some extent, respectively. To further describe the effectiveness of the microscopic method quantitatively, the failure strength comparison is shown in Table 3. A good consistency can be found, and the maximum relative error is equal to 5.51% when the composite laminate ${\left[0/90\right]}_{4s}$ is considered at an ambient temperature of 100 °C.

#### 3.2.2. Proposed Fatigue Damage Model

The stress amplitude versus number of cycles (*S-N*) curve is one of the most popular methods to describe fatigue life in the engineering field. In order to effectively predict the service life of composite structures, a new set of fatigue equations based on the computational micromechanics’ RVE is proposed. Here, AS4 fibers were considered to be linearly degenerated with respect to the cycling number. Matrix fatigue can be described by Chaboch’s fatigue equation [46,47,48] as shown in Table 4. It indicates that the fatigue life, $N_{f}$, is a function of the maximum stress amplitude, $\sigma_{\max}$, and average stress amplitude, $\bar{\sigma }$. $\sigma_{u}$ and $\sigma_{fl}$ indicate the maximum stress and fatigue limit of the polymer matrix. The fatigue parameters, a, β, and, will be confirmed according to the *S-N* curve of the PEEK matrix and composites. It can easily be found that this fatigue equation is restricted to the loading condition. If fatigue tests were executed with a stress ratio of $\sigma_{\min}^{}/\sigma_{\max}=0.3$, the denominator, $\sigma_{\max}-\bar{\sigma }-\sigma_{fl}$, may be a negative number. To extend its application range, a new fatigue pertinent equation is proposed as shown in Table 4. Here, the parameter, w, is defined as a fatigue control parameter, which can be acquired according to the fatigue experimental data of the matrix and composite materials.

## 4. Discussions 

### 4.1. Fatigue Model Validation

**Figure 5:**
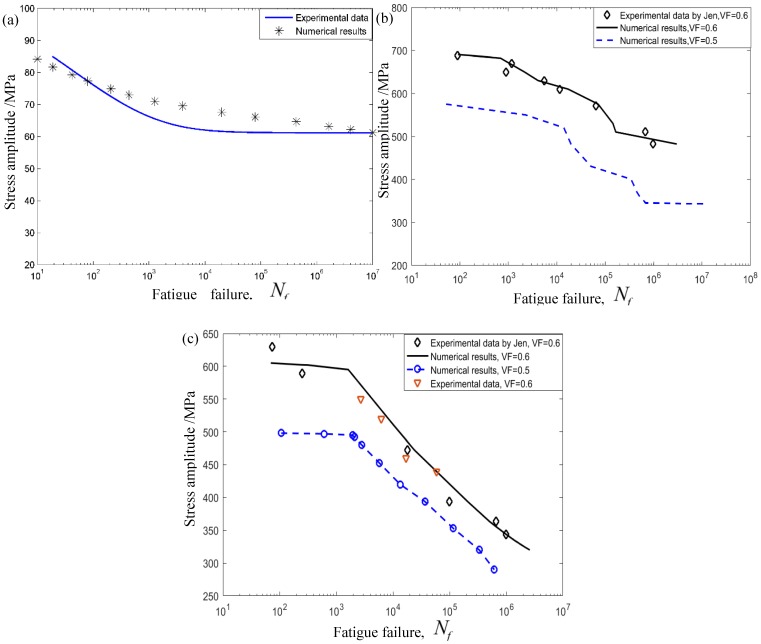
Comparisons of fatigue life at room temperature. (**a**) PEEK matrix at room temperature. (**b**) Composite laminates ${\left[0/45/90/-45\right]}_{4s}$ at room temperature. (**c**) Composite laminates ${\left[0/45/90/-45\right]}_{4s}$ at 100 °C.

In the fatigue test, fatigue properties of constituent materials were closely related to the loading number and amplitude. Based on the tested results by Zhu [16], the fatigue limit of 1300 MPa of AS4 fibers was considered in the example. According to the fatigue test S-N curves of the PEEK matrix at room temperature, as well as AS4/PEEK composites at room and elevated temperatures executed by McKeen et al. [49], the fatigue limit of 61.21 and 21.47 MPa of the PEEK matrix at room temperature and 100 °C were employed in predicting the service life of composites, respectively. Here, the MTS was employed to execute T-T fatigue loading, and the environment chamber was employed to execute fatigue tests at 100 °C as a further comparison. A constant stress amplitude, by controlling the load mode with the stress ratio of $\sigma_{\min}/\sigma_{\max}=0.1$, was employed, and the frequency was 2 Hz. According to the experimental curves of the PEEK and composites at room temperature [49] as shown in Figure 5a,b, the fatigue parameters in the proposed fatigue equation can be confirmed as follows: β=6, a=0.06, M=145 MPa and w=0.01. It can easily be seen that the theoretical prediction shows good consistency with the experimental data at room temperature, and a higher cycling loading will sharply decrease their fatigue endurance. It is interesting to mention that similar conclusions were found at 100 °C as shown in Figure 5c when the identical fatigue parameters mentioned above were employed in the example. In addition, the raised ambient temperature tended to reduce their service life to a great degree. 

To further illuminate the VF influences, a different volume fraction of reinforced fibers of 0.5 was also considered in the example. From the figure, it is shown that the higher VF tended to increase their service life at both room and elevated temperatures. It means that AS4 fibers play an important role in increasing the fatigue life and failure strength for composite structures. In detail, the fatigue limits were 578.59 and 342.12 MPa when the VF of reinforced fibers was 0.6 and 0.5, respectively.

### 4.2. Evaluations of Fatigue Life under Coupled Thermo–Mechanical Loading

**Figure 6:**
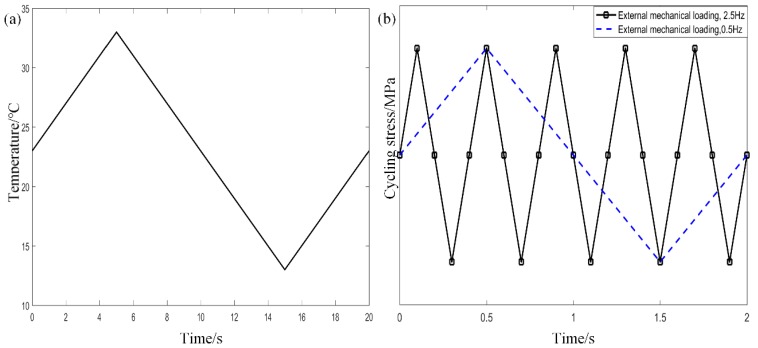
Loading modes on composites. (**a**) Temperature cycling. (**b**) Two typical mechanical loadings.

The material parameters are always temperature dependent. To reveal coupled thermo–mechanical effects on stiffness degeneration and fatigue failure properties, a small temperature variation between 13 and 33 °C was investigated. In the study, a temperature cycling frequency of 0.05 Hz was considered as shown in Figure 6a. For comparisons, two T-C mechanical loadings of 0.5 and 2.5 Hz were considered as shown in Figure 6b. 

**Figure 7:**
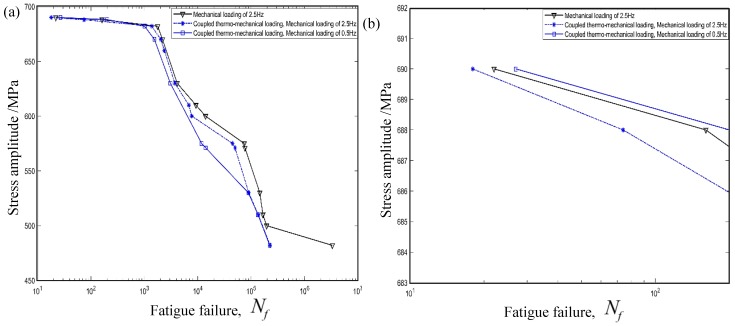
Fatigue life of composite laminates under thermo-mechanical loading at room temperature. (**a**) Temperature cycling effects. (**b**) Partial enlargement graph.

Figure 7a indicates the stress amplitude of ${\left[0/45/90/-45\right]}_{4s}$ composite laminates with respect to the cycling number in coupled thermo–mechanical loading. It can easily be seen that temperature cycling coupled with a mechanical loading of 2.5 Hz tends to sharply decrease the fatigue life of composite structures. However, a different conclusion was obtained when a mechanical frequency of 0.5 Hz was considered in the example. In detail, it is interesting to mention that coupled temperature cycling tends to firstly increase and then decrease the fatigue life with a decrease of the stress amplitude. To further clearly discern this phenomenon, a local enlargement graph was employed as shown in Figure 7b. In detail, the temperature cycling tends to extend the fatigue life of composite structures when the stress amplitude derived from mechanical loading is higher than 686.3 MPa. However, an opposite conclusion was acquired once a small mechanical amplitude was considered. That is, the temperature cycling tends to decrease the fatigue life when a small stress amplitude was considered. In addition, the cycling frequency of mechanical loading effects on the fatigue life of composite laminates under the thermo–mechanical loading can be ignored when the stress amplitude is less than 529.9 MPa.

### 4.3. Local Stress Distribution under Coupled Thermo–Mechanical Loading

To reveal the fatigue failure mechanisms under coupled thermo–mechanical loading, it is critical to accurately capture stress distributions at the microscopic scale. Based on the failure and fatigue validations mentioned above, herein, local stress fields in the RVE were employed. In the following cases, it should be noted that the frequency ratio of 0.1 of temperature cycling vs. mechanical cycling was considered. Meanwhile, the mechanical loading ratio of $\sigma_{\min}/\sigma_{\max}$ was −1.

**Figure 8:**
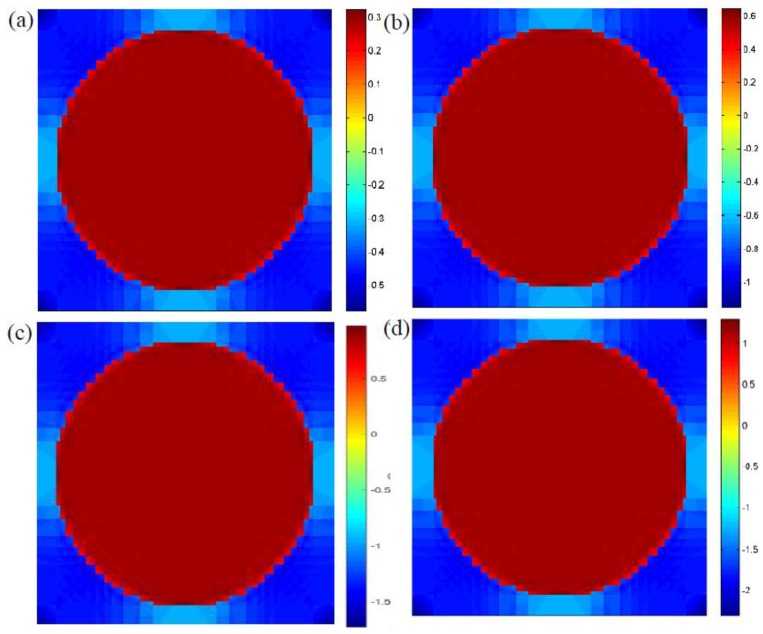
Microscopic stress distributions $\sigma_{11}$ of the RVE under different ambient temperatures: (**a**) 25 °C, (**b**) 27 °C, (**c**) 29 °C, (**d**) 31 °C.

**Figure 9:**
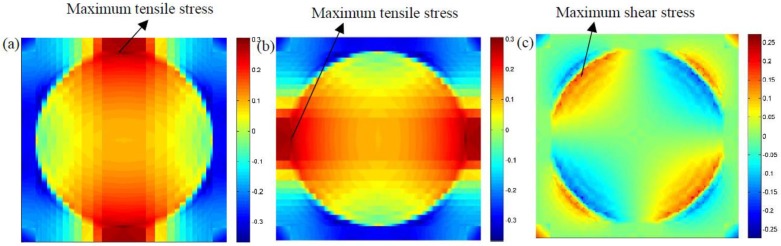
Microscopic stress distributions of the composites subjected to temperature variations at 5 s (**a**) $\sigma_{22}$ (**b**) $\sigma_{33}$ (**c**) $\sigma_{23}$.

Figure 8 indicates the longitudinal stress distributions due to a rising temperature from 25 to 31 °C. It can easily be found that a higher temperature tends to increase the maximum stress, $\sigma_{11}$, at the microscopic scale, and the thermal stress exhibits compression in the matrix and tension in fibers due to the mismatch of thermal expansion coefficients between reinforced fibers and the matrix. In addition, the maximum compressive stress was found in the matrix materials. Figure 9 indicates the microscopic stress distributions of $\sigma_{22}$,$\sigma_{33}$, and $\sigma_{23}$ subjected to a temperature loading at 33 °C. It was observed that the maximum tensile and shear stresses are both located at the interface between the fibers and matrix materials.

**Figure 10:**
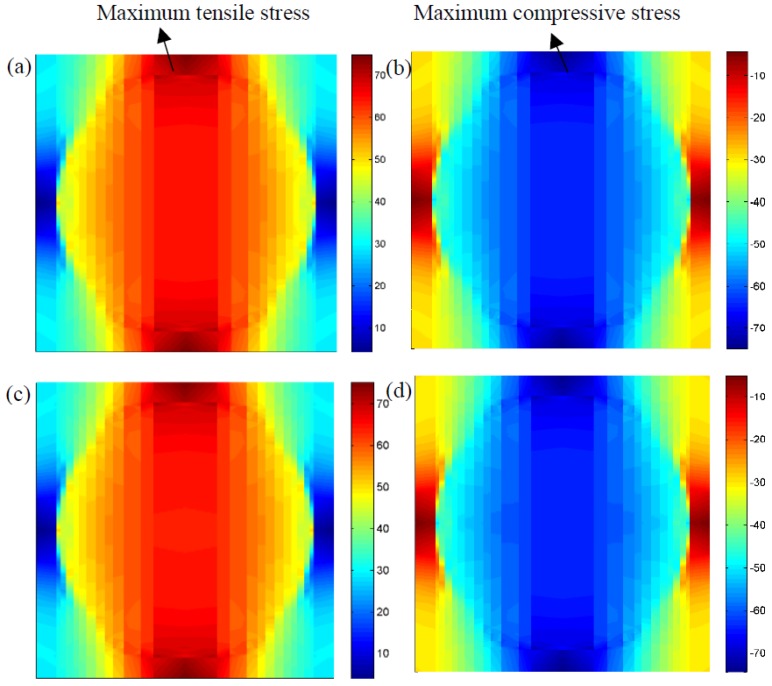
Microscopic stress distributions, $\sigma_{22}$, of the composites (**a**) without temperature variation at 0.5 s, (**b**) without temperature variation at 1.5 s, (**c**) with temperature variation at 0.5 s, (**d**) with temperature variation at 1.5 s.

Figure 10a,b indicates the microscopic stress distributions, $\sigma_{22}$, of composites under the pure mechanical loadings at 0.5 and 1.5 s, respectively. It can be seen from the figures that the maximum stresses are located at the interface between the matrix materials and fibers, and a distinct difference of stress distributions can easily be found. In detail, the reinforced fibers, which support the tensile or compressive loadings, are decided by the external loading status. To quantitatively explain coupled cycling temperature effects on their effective properties, cycling temperatures at 0.5 s and 1.5 s were also considered as shown in Figure 10c,d. Similarly, it can also be found that the maximum stress at the interface region is higher than that in the fiber or matrix region. In other words, this finite cycling temperature is insufficient to alter the stress distributions and maximum stress region. In addition, it was revealed that the maximum tensile stress of the composites under mechanical loading at 0.5 s was 74.849 MPa as shown in Figure 10a. While a higher stress of 75.129 MPa was found at 0.5 s when the cycling temperature was considered as shown in Figure 10c. This slight increase in maximum stress may be increase the probability of the initial damages at the interface with composites. 

**Figure 11:**
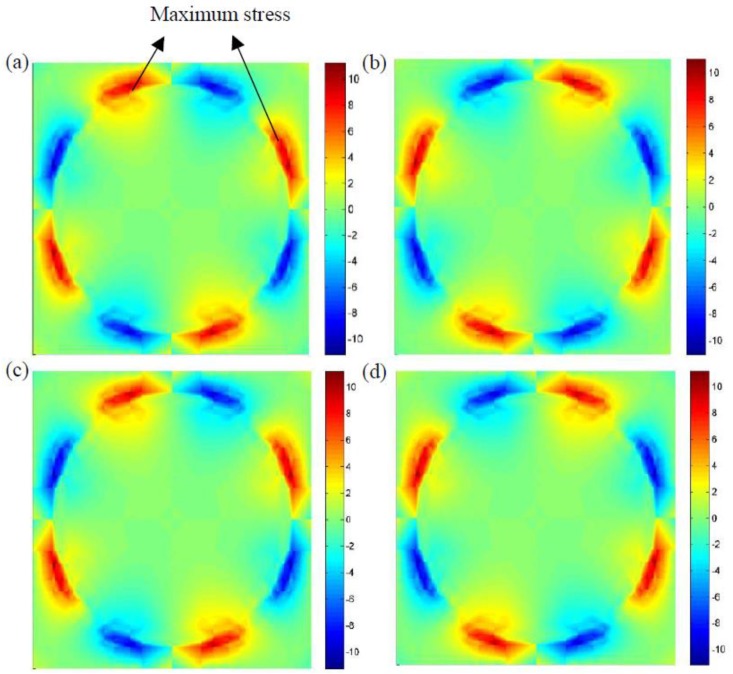
Microscopic stress distributions, $\sigma_{23}$, of the composites (**a**) with temperature variation at 0.5 s, (**b**) without temperature variation at 1.5 s, (**c**) with temperature variation at 0.5 s, and (**d**) with temperature variation at 1.5 s.

Figure 11a,b indicates the microscopic stress distributions, $\sigma_{23}$, under the mechanical loading at 0.5 and 1.5 s, respectively. Similar to the stress distributions, $\sigma_{22}$, mentioned above, it can be seen that the maximum shear stress, $\sigma_{23}$, is also located at the interface region, and the temperature cycling will not affect the microscopic stress distributions as shown in Figure 11c,d. In addition, it can be found that a higher temperature tends to increase the maximum shear stress in composites. In detail, when a loading time of 0.5 s was considered, the maximum shear stresses were 11.174 and 11.218 MPa under mechanical loading and coupled thermo–mechanical loading, respectively. 

## 5. Conclusions

This study focused on the fatigue life of composite structures under coupled thermo–mechanical loading. To validate the proposed microscopic model and the chosen RVE, the prediction results of effective moduli and failure properties of composites at room and elevated temperatures were both investigated and compared with experimental data. It was shown that the maximum relative error of the failure strength was equal to 5.51% when the composite laminate ${\left[0/90\right]}_{4s}$ was considered at an ambient temperature of 100 °C. In order to extend the application range of the fatigue model, the control parameter, u, was introduced. Compared with experimental data at room temperature and 100 °C, it was shown that the proposed fatigue equation can be effectively employed to describe the fatigue life. A higher VF tends to sharply increase their service life at both room and elevated temperatures.

It is interesting to mention that thermal cycling will deeply affect their service life. When a mechanical frequency of 0.5 Hz was considered, the coupled temperature cycling tended to first increase and then decrease the fatigue life of composites with the decreasing of stress amplitude. However, the finite cycling temperature studied in this paper was insufficient to alter the stress distributions, and the maximum stress was located at the interphase between reinforced fibers and matrix materials. The proposed method in this paper is also suitable for on-line life evaluation of composite structures of whether the loads can be measured exactly by the strain sensors. However, a higher accuracy of the microscopic stress field in the RVE can be acquired when parameter elements are considered as discrete to the RVE at the microscopic scale. Therefore, our research group is trying to construct parameter elements to achieve an effective life evaluation with a higher accuracy. In addition, the SEM will be used to further reveal failure mechanisms of composites at the elevated temperature.

## Figures and Tables

**Table 1:** Material parameters of PEEK matrix at room temperature and elevated temperature.

PEEK	Elastic Modulus (GPa)	Poisson’s Ratio	Tensile Strength *X*_T_ (MPa)	Compressive Strength *X*_c_ (MPa)	Shear Strength *S*_m_ (MPa)	Thermal Coefficient *α*_2_ (10^−6^/Cº)	Thermal Conductivity (W/(m °L))
23 °C	3.64	0.4	96.58	118	53.0	47.0	0.163
100 °C	1.52	0.4	50.37	-	14.43	47.0	0.231

**Table 2:** Longitudinal moduli study of composite laminates.

Temperature	23 °C (Room Temperature)		100 °C	
Layer Sequence	${\left[0^{{}^{\circ}}/90^{{}^{\circ}}\right]}_{4s}$	${\left[0^{{}^{\circ}}/+45^{{}^{\circ}}/90^{{}^{\circ}}/-45^{{}^{\circ}}\right]}_{2s}$	${\left[0^{{}^{\circ}}/90^{{}^{\circ}}\right]}_{4s}$	${\left[0^{{}^{\circ}}/+45^{{}^{\circ}}/90^{{}^{\circ}}/-45^{{}^{\circ}}\right]}_{2s}$
Experimental Data (GPa)	77.68 ± 2.94	55.01 ± 1.68	75.23 ± 2.71	52.09 ± 1.61
Theoretical Results (GPa)	74.33	51.81	72.87	50.92

**Table 3:** Finial failure strength comparisons of composite laminates.

Temperature	23 °C		100 °C	
Layer sequence	${\left[0^{{}^{\circ}}/90^{{}^{\circ}}\right]}_{4s}$	${\left[0^{{}^{\circ}}/+45^{{}^{\circ}}/90^{{}^{\circ}}/-45^{{}^{\circ}}\right]}_{2s}$	${\left[0^{{}^{\circ}}/90^{{}^{\circ}}\right]}_{4s}$	${\left[0^{{}^{\circ}}/+45^{{}^{\circ}}/90^{{}^{\circ}}/-45^{{}^{\circ}}\right]}_{2s}$
Experimental data (MPa)	1035.2	708.9	859.7	629.52 (100 °C)
Theoretical results (MPa)	1031.5907	696.0	907.1	604.8

**Table 4:** A comparison between Chaboch’s fatigue equation and the improved fatigue equation.

Chaboch’s Equation	Proposed Fatigue Pertinent Equation
$N_{f}=\frac{(\sigma_{u}-\sigma_{\max}){\left(\frac{M}{\sigma_{\max}-\bar{\sigma }}\right)}^{\beta }}{a(1+\beta )(\sigma_{\max}-\bar{\sigma }-\sigma_{fl})}$	$N_{f}(T)=\frac{(\sigma_{u}(T)-\sigma_{\max}){\left(\frac{M}{\sigma_{\max}-w\bar{\sigma }}\right)}^{\beta }}{a(1+\beta )(\sigma_{\max}-\sigma_{fl}(T))}$
